# Associations between Inadequate Parenting Practices and Behavioral Problems in Children and Adolescents with Attention Deficit Hyperactivity Disorder

**DOI:** 10.1155/2015/683062

**Published:** 2015-12-30

**Authors:** Maria Cristina Triguero Veloz Teixeira, Regina Luisa de Freitas Marino, Luiz Renato Rodrigues Carreiro

**Affiliations:** Universidade Presbiteriana Mackenzie, 01302-907 São Paulo, SP, Brazil

## Abstract

Children and adolescents with ADHD present behaviors such as impulsiveness, inattention, and difficulties with personal organization that represent an overload for parents. Moreover, it also increases their level of stress and leads them to resort to inadequate educational strategies. The present study verifies associations between inadequate parenting practices and behavioral profiles of children and adolescents with ADHD. The sample was composed of 22 children with ADHD (age range 6–16 years) and their mothers. Spearman correlation analyses were made with the scores of Parenting Style Inventory (PSI) and Child Behavior Checklist for ages 6–18 (CBCL/6–18). Results indicate statistically significant associations between behavioral problems and the use of punishment practices and negligence. When assessing a child with ADHD, it is important to verify the predominant types of parenting practices that can influence both immediate interventions and the prognosis of the disorder.

## 1. Introduction

Attention Deficit Hyperactivity Disorder (ADHD) is a neurodevelopmental disorder characterized by a persistent pattern of inattention and/or hyperactivity-impulsiveness that affects significantly the social and academic functioning of the child [1]. Inadequate patterns of family interaction and parenting practices in childhood can influence the course of the disorder, aggravate its signs and symptoms, and contribute to the secondary development of other behavioral problems, such as oppositional defiant disorder and aggressiveness [2, 3].

According to parental perception, children with ADHD, when compared to children without the disorder, demand more efforts to be managed and directed to the acquisition of adjusted behavioral repertoires and present less capacity to regulate their behaviors [4, 5]. They also present more sleeping problems and emotional and motivational difficulties, especially those with predominance of hyperactive-impulsive symptoms [6]. Due to abnormalities of neural networks involving prefrontal cortex of children with ADHD [7], they usually show impaired executive functions, such as difficulties in inhibitory control, planning, and working memory [8]. These factors represent an overload for parents, as these children are more dependent on parental external feedback to regulate their behavior [9]. The fact that children with ADHD often depend on external parental guidelines interferes in several aspects of the family routine. Previous studies mention that, compared to parents of children without the disorder, some parents (especially mothers) of children with ADHD work fewer hours or quit their jobs to have more time to look after the child and help him/her with school activities and daily routine [10, 11].

Parents of children with ADHD more often report family conflicts [12]; disorganization [4]; less perception of family support [13]; and less adherence to rules and preestablished routine, mainly in adolescence [14]. Typical behaviors displayed by children and adolescents with ADHD such as impulsiveness, agitation, inattention [15, 16], difficulties with personal organization, and adapting to routine in the family environment [14], among others, represent an overload to parents and increase the levels of parental stress [12]. Psychological and physical overload for parents often lead to the use of inappropriate parenting practices [17] as an attempt to reduce behaviors which are considered disruptive, many of them typical for children with ADHD of the hyperactive-impulsive type [14, 18]. Previous studies have verified the association between externalizing and internalizing behavioral problems in children and adolescents and parenting practices [19, 20], especially those characterized by negligence, parental rejection, lack of discipline [21], and inconsistent punishment [22]. Thus, improving parenting skills through behavioral training appears as an effective intervention for children with ADHD [23].

Another situation that can aggravate behavioral problems is the lack of or delay in providing proper intervention for children with a psychiatric disorder such as ADHD. In Latin American countries such as Mexico, in population cohorts of adolescents aged 12 to 17 years, it was verified that 1 out of 7 individuals with psychiatric disorders (including ADHD) did not have access to mental health services [24]. In developed countries, the existence of public health services does not always guarantee that they are broadly accessible, considering the intervenience of factors such as place of residence and financial conditions of the users [25, 26]. In Brazil, a lack of mental health services for children and adolescents was verified, while the existing specialized services are still not enough and unequally distributed [27]. The present study aims to verify associations between inadequate parenting practices (negligence, physical abuse, and inconsistent punishment) and behavioral profiles of children and adolescents with ADHD.

## 2. Material and Method

### 2.1. Participants

The sample was selected from a neuropsychological, behavioral, and clinical assessment protocol involving children and adolescents with complaint of inattention and hyperactivity, which was carried out by a national reference center that identifies signs compatible with ADHD in the community [28]. The protocol assessed 138 children and adolescents between 2014 and 2015. Initially, 52 of them were diagnosed with ADHD, but only 22 parents agreed to fill in the Parenting Style Inventory. The sample was thus composed of 22 children and adolescents with ADHD (6–16 years; M = 9,77; SD = 2,91), enrolled from 1st grade of elementary school to 1st grade of high school and their mothers (in the condition of main caregiver). All participants were assessed by the same child neurologist who established the ADHD diagnosis based on DSM-IV clinical criteria [29], with predominance of combined type. Children and adolescents were assessed to exclude psychiatric disorders and cognitive deficits through Wechsler Intelligence Scale for Children (WISC-III) [30]. The study was approved by the Ethics Committee in Research Involving Humans of Universidade Presbiteriana Mackenzie, São Paulo, Brazil (CEP/UPM number 1232/04/2010 and CAAE number 0039.0.272.000-10). Participants were informed about their voluntary right to participate in this study and a written consent was obtained prior to data collection.

### 2.2. Instruments

#### 2.2.1. Parenting Style Inventory (PSI)

The inventory assesses seven parenting practices, two of them positive (positive monitoring and moral behavior) and five negative (negative monitoring, inconsistent punishment, discipline absence, negligence, and physical abuse). It consists of 42 items to be responded by parents about strategies adopted to orient the child [31]. For this study the following practices were considered: (a) negligence: the parent does not meet the child's emotional or practical needs and is absent from parental responsibilities; (b) inconsistent punishment: the parent punishes or reinforces a behavior according to his/her mood and not to contingencies; (c) physical abuse: the parent uses physical strength to hurt or bruise the child on the grounds of educating them.

#### 2.2.2. Child Behavior Checklist for Ages 6–18 (CBCL/6–18)

The inventory was responded by parents considering the behavior of their children in the previous six months. It is composed of 136 items and is divided into two parts. The first part assesses social competence/adaptive functioning, and the second part identifies behavioral problems grouped in the syndromes scales, DSM-oriented scales, internalizing, externalizing, and total behavior problems scales. This study considered only behavioral problems of syndromes, externalizing, internalizing, and total problems scales [32, 33].

### 2.3. Data Analysis Procedures

Spearman correlation analyses were made with the scores from both instruments to verify associations between parenting practices (negligence, inconsistent punishment, and physical abuse) and behavioral problems in children with ADHD.

## 3. Results and Discussion

Results of correlation analyses among the studied variables can be seen in Table 1. The scale Attention Deficit/Hyperactivity Problems of CBCL covers behaviors such as “cannot sit still, restless, or hyperactive,” “fails to finish things he/she starts,” and “impulsive or acts without thinking.” Correlations coefficients indicate positive and statistically significant associations with parenting practices of physical abuse and inconsistent punishment. Parents of children with inattention and hyperactivity symptoms are more likely to resort to practices of physical abuse and inconsistent punishment [34, 35]. According to Hadianfard [36], children with ADHD are more likely to be victims of violent parenting practices, especially psychological abuse and negligence, when compared to children without the disorder. Mothers of children with ADHD of the hyperactive type employ physical disciplinary practices more frequently than mothers of children with ADHD of combined or inattentive types or mothers of children without the disorder. Hyperactivity seems to be related to an experience of increased stress by parents. This is caused by the difficulty to manage this behavior and favors the employment of coercive practices in an attempt to immediately control the child's hyperactive behavior [37].

Individuals who suffer physical abuse and those who present symptoms of ADHD are alike in some aspects of behavioral expression. Both present tendency to externalizing behavioral problems, such as aggressiveness and opposition, and to internalizing problems, such as social withdrawal and peer rejection. Children with ADHD who suffer physical abuse are perceived by parents, teachers, and peers as excessively aggressive [38]. Some items of the Aggressiveness scale of CBCL are “destroys his/her own things,” “destroys things belonging to his/her family and others,” “gets in many fights,” and “physically attacks people.” As it can be seen in Table 1, these problems were positively correlated with inconsistent punishment and physical abuse. Previous study indicated that parents of children with ADHD are more prone to use abusive discipline methods. In addition, parents' abusive discipline methods are associated with increase of the child's behavioral problems [34].

Other behavioral problems of the Rule-Breaking Behavior scale (CBCL/6–18), such as “does not seem to feel guilty after misbehaving,” “lying or cheating,” “disobedient at home, school, or other places,” and “steals outside the home,” present positive correlation with abuse and physical maltreatment. In the sphere of internalizing problems, physical abuse was also correlated to items of Thought Problems scale, such as “nervous movements, twitching,” “cannot get his/her mind off certain thoughts; obsessions,” “repeats certain actions over and over,” and “strange behavior.” These problems could be exacerbated by the influence of several factors, including the use of parenting abusive practices, as shown by previous studies [39, 40].

The perception of mothers about the academic competences of their children has also presented statistically significant associations with behavioral problems, as it was the case with inadequate parenting practices. The School scale of CBCL (Table 1) had a negative correlation with the practice of negligence. This indicates that increased use of this practice is associated with a reduction of academic performance. Parents that help with school tasks contribute to the development of a study-related behavioral repertoire and favor the learning process [41]. Thus, the absence of parental involvement in such activities, typical of negligence, can contribute to the child's poor performance, the increase of behavioral problems, and worsening of ADHD's typical symptoms. According to Gonida and Cortina [41], parental engagement with school activities brings school and home closer and promotes the development of academic skills and specific knowledge. Beliefs of parents about the academic performance of their children have an important role in motivation and performance [42]. The less the parents believe in the success of their children, the less the children themselves believe in their capacity to have a good academic performance [41]. Together with negligence, Osofsky [43] shows that physical abuse is also related to poor academic performance and even to delays in cognitive development.

## 4. Conclusion

The findings of the present study indicate that the parents of the 22 assessed children keep an expressive use of parenting practices characterized by punishment and negligence. Such strategies are associated with several patterns of behavioral problems, including those typical of ADHD, but also with other emotional and behavioral problems, mainly of the externalizing type. Children and adolescents with ADHD often find themselves under immediate effect of several adverse factors that can aggravate the behavioral symptoms of the disorder as well as favoring the onset of other psychiatric problems and symptoms. Some examples are excessive use of physical punishment, yelling, absence of parents performing the role of caregiver, and lack of positive parenting practices, that is, adequate use of rules and instructions, establishment of routine, and positive stimulation of adequate behaviors. Other variables that could potentially work as intermediate factors of inadequate parenting practices, such as the caregiver's mental health, were not controlled.

Correlation studies indicate associations among variables, but not their cause and effect relationships. The way a child behaves can influence the adoption of a certain parenting style as well as certain parenting practices that, in their turn, can have short- and long-term negative influence on the child's behavior. This phenomenon is described as a vicious cycle between behavioral problems and parenting practices. The present results suggest that the general clinical assessment of children and adolescents with ADHD should consider parenting practices to better understand mental health problems among this population. We highlight the importance of including the parents of children with ADHD in the intervention process, in order to provide orientation on the management of behavioral problems and thus attempt to minimize the aforementioned vicious cycle.

## Figures and Tables

**Table 1:** Statistically significant correlations between inadequate parenting practices and behavioral problems in children with ADHD.

Parenting Style Inventory/parenting practices	CBCL/6–18	Spearman's rho	*p*	*n*
Inconsistent punishment	Anxious/depressed	0,24	0.284	22
	Withdrawn/depressed	−0,09	0.695	22
	Somatic complaints	−0,06	0.791	22
	Social problems	0,23	0.303	22
	Thought Problems	0,21	0.355	22
	Attention and hyperactivity problems	0,49^*∗*^	0.020	22
	Rule-Breaking Behavior	0,21	0.345	22
	Aggressive behavior	0,46^*∗*^	0.030	22
	Internalizing problems	0,17	0.446	22
	Externalizing problems	0,47^*∗*^	0.024	22
	Total problems	0,33	0.134	22
	Activities	−0,13	0.575	22
	Social competence	−0,19	0.394	22
	School	−0,17	0.463	21
	Total competence	−0,14	0.533	21

Negligence	Anxious/depressed	−0,09	0.683	22
	Withdrawn/depressed	0,02	0.935	22
	Somatic complaints	−0,32	0.141	22
	Social problems	−0,08	0.732	22
	Thought Problems	−0,01	0.963	22
	Attention and hyperactivity problems	0,38	0.078	22
	Rule-Breaking Behavior	0,22	0.336	22
	Aggressive behavior	−0,24	0.280	22
	Internalizing problems	−0,13	0.558	22
	Externalizing problems	−0,17	0.442	22
	Total problems	−0,09	0.682	22
	Activities	−0,36	0.102	22
	Social competence	−0,23	0.307	22
	School	−0,48^*∗*^	0.025	21
	Total competence	−0,42	0.058	21

Physical abuse	Anxious/depressed	0,20	0.373	22
	Withdrawn/depressed	−0,18	0.427	22
	Somatic complaints	−0,15	0.519	22
	Social problems	0,41	0.058	22
	Thought Problems	0,42^*∗*^	0.048	22
	Attention and hyperactivity problems	0,56^*∗∗*^	0.006	22
	Rule-Breaking Behavior	0,48^*∗*^	0.021	22
	Aggressive behavior	0,74^*∗∗*^	<0.001	22
	Internalizing problems	0,10	0.668	22
	Externalizing problems	0,72^*∗∗*^	<0.001	22
	Total problems	0,55^*∗∗*^	0.008	22
	Activities	−0,33	0.137	22
	Social competence	−0,37	0.089	22
	School	−0,15	0.505	21
	Total competence	−0,42	0.055	21

Note: the social competence scale was not responded by one child of the sample.

^*∗∗*^Significant correlation at the 0.01 level

^*∗*^Significant correlation at the 0.05 level.

## References

[1] American Psychiatric Association (2013). *Diagnostic and Statistical Manual of Mental Disorders (DSM-5)*.

[2] Wang C. H., Mazursky-Horowitz H., Chronis-Tuscano A. (2014). Delivering evidence-based treatments for child attention-deficit/hyperactivity disorder (ADHD) in the context of parental ADHD. *Current Psychiatry Reports*.

[3] Tung I., Brammer W. A., Li J. J., Lee S. S. (2015). Parenting behavior mediates the intergenerational association of parent and child offspring adhd symptoms. *Journal of Clinical Child and Adolescent Psychology*.

[4] Schroeder V. M., Kelley M. L. (2009). Associations between family environment, parenting practices, and executive functioning of children with and without ADHD. *Journal of Child and Family Studies*.

[5] Leijten P., Raaijmakers M. A., Orobio de Castro B., van den Ban E., Matthys W. (2015). Effectiveness of the incredible years parenting program for families with socioeconomically disadvantaged and ethnic minority backgrounds. *Journal of Clinical Child & Adolescent Psychology*.

[6] Gong J., Yuan J., Wang S., Shi L., Cui X., Luo X. (2014). Feedback-related negativity in children with two subtypes of attention deficit hyperactivity disorder. *PLoS ONE*.

[7] Bush G. (2010). Attention-deficit/hyperactivity disorder and attention networks. *Neuropsychopharmacology*.

[8] O'Brien J. W., Dowell L. R., Mostofsky S. H., Denckla M. B., Mahone E. M. (2010). Neuropsychological profile of executive function in girls with attention-deficit/hyperactivity disorder. *Archives of Clinical Neuropsychology*.

[9] Barkley R. A. (1997). *ADHD and the Nature of Self-Control*.

[10] Sharif F., Zarei S., Shooshtari A. A., Vossoughi M. (2015). The effect of stress management program using cognitive behavior approach on mental health of the mothers of the children with attention deficit hyperactivity disorder. *Iranian Journal of Pediatrics*.

[11] Kvist A. P., Nielsen H. S., Simonsen M. (2011). The effects of children's ADHD on parents' relationship dissolution and labor supply. *IZA Discussion Paper*.

[12] Wiener J., Biondic D., Grimbos T., Herbert M. (2015). Parenting stress of parents of adolescents with attention-deficit hyperactivity disorder. *Journal of Abnormal Child Psychology*.

[13] Moghaddam M. F., Assareh M., Heidaripoor A., Rad R. E., Pishjoo M. (2013). The study comparing parenting styles of children with ADHD and normal children. *Archives of Psychiatry and Psychotherapy*.

[14] Modesto-Lowe V., Chaplin M., Godsay V., Soovajian V. (2014). Parenting teens with attention-deficit/hyperactivity disorder: challenges and opportunities. *Clinical Pediatrics*.

[15] Modesto-Lowe V., Danforth J. S., Brooks D. (2008). ADHD: does parenting style matter?. *Clinical Pediatrics*.

[16] Theule J., Wiener J., Tannock R., Jenkins J. M. (2013). Parenting stress in families of children with ADHD: a meta-analysis. *Journal of Emotional and Behavioral Disorders*.

[17] Gomide P. I. C., Salvo C. G., Pinheiro D. P. N., Sabbag G. M. (2005). Correlação entre práticas educativas, depressão, estresse e habilidades sociais. *Psico-USF*.

[18] Robin A. L. (2014). Family therapy for adolescents with ADHD. *Child and Adolescent Psychiatric Clinics of North America*.

[19] Bolsoni-Silva A. T., Del Prette A. (2003). Problemas de Comportamento: um panorama da área. *Revista Brasileira de Terapia Comportamental e Cognitiva*.

[20] Cia F., Barham E. J., Fontaine A. M. G. V. (2010). Impactos de uma intervenção com pais: o desempenho acadêmico e comportamento das crianças na escola. *Psicologia: Reflexão e Crítica*.

[21] Nunes S. A. N., Faraco A. M. X., Vieira M. L., Rubin K. H. (2013). Externalizing and internalizing problems: contributions of attachment and parental practices. *Psicologia: Reflexão e Crítica*.

[22] Tung I., Lee S. S. (2014). Negative parenting behavior and childhood oppositional defiant disorder: differential moderation by positive and negative peer regard. *Aggressive Behavior*.

[23] Fabiano G. A., Schatz N. K., Aloe A. M., Chacko A., Chronis-Tuscano A. (2015). A systematic review of meta-analyses of psychosocial treatment for attention-deficit/hyperactivity disorder. *Clinical Child and Family Psychology Review*.

[24] Borges G., Benjet C., Medina-Mora M. E., Orozco R., Nock M. (2008). Suicide ideation, plan, and attempt in the Mexican adolescent mental health survey. *Journal of the American Academy of Child & Adolescent Psychiatry*.

[25] De Voursney D., Sondheimer D., Drumm A. (2013). Improving community-based mental health care for children: a commentary. *Administration and Policy in Mental Health and Mental Health Services Research*.

[26] Paananen R., Santalahti P., Merikukka M., Rämö A., Wahlbeck K., Gissler M. (2013). Socioeconomic and regional aspects in the use of specialized psychiatric care—a finnish nationwide follow-up study. *European Journal of Public Health*.

[27] Paula C. S., Lauridsen-Ribeiro E., Wissow L., Bordin I. A. S., Evans-Lacko S. (2012). How to improve the mental health care of children and adolescents in Brazil: actions needed in the public sector. *Revista Brasileira de Psiquiatria*.

[28] Carreiro L. R. R., Schwartzman J. S., Cantiere C. N. (2014). Protocolo interdisciplinar de avaliação neuropsicológica, comportamental e clínica para crianças e adolescentes com queixas de desatenção e hiperatividade. *Revista Psicologia: Teoria e Prática*.

[29] American Psychiatric Association (2000). *Diagnostic and Statistical Manual of Mental Disorders (DSM-IV-TR)*.

[30] Wechsler D. (2002). *Escala de Inteligência Wechsler para Crianças (WISC-III)*.

[31] Gomide P. I. C. (2006). *Inventário de Estilos Parentais—IEP: Modelo teórico, manual de aplicação, apuração e interpretação*.

[32] Achenbach T. M., Rescorla L. A. (2001). *Manual for the ASEBA School-Age Forms Profiles*.

[33] Bordin I. A., Rocha M. M., Paula C. S. (2013). Child behavior checklist/CBCL, youth self-report/YSR and teacher's report form/TRF: an overview of the development of original and Brazilian version. *Cadernos de Saúde Pública*.

[34] Evinç Ş. G., Gençöz T., Foto-Özdemir D., Akdemir D., Karadag F., Ünal F. (2014). Child maltreatment and associated factors among children with ADHD: a comparative study. *The Turkish Journal of Pediatrics*.

[35] Ellis B., Nigg J. (2009). Parenting practices and attention-deficit/hyperactivity disorder: new findings suggest partial specificity of effects. *Journal of the American Academy of Child & Adolescent Psychiatry*.

[36] Hadianfard H. (2014). Child abuse in group of children with attention deficit-hyperactivity disorder in comparison with normal children. *International Journal of Community Based Nursing and Midwifery*.

[37] Weinberger K. A., Gardner D. M., Gerdes A. C. (2015). Maternal functioning differences based on ADHD subtype. *Journal of Attention Disorders*.

[38] Briscoe-Smith A. M., Hinshaw S. P. (2006). Linkages between child abuse and attention-deficit/hyperactivity disorder in girls: behavioral and social correlates. *Child Abuse and Neglect*.

[39] Zwi M., Jones H., Thorgaard C., York A., Dennis J. A. (2011). Parent training interventions for Attention Deficit Hyperactivity Disorder (ADHD) in children aged 5 to 18 years. *Cochrane Database of Systematic Reviews*.

[40] Ford J. D., Racusin R., Ellis C. G. (2000). Child maltreatment, other trauma exposure, and posttraumatic symptomatology among children with oppositional defiant and attention deficit hyperactivity disorders. *Child Maltreatment*.

[41] Gonida E. N., Cortina K. S. (2014). Parental involvement in homework: relations with parent and student achievement-related motivational beliefs and achievement. *British Journal of Educational Psychology*.

[42] Eccles J. S., Grusec J. E., Hastings P. D. (2007). Families, schools, and developing of achievement-related motivations and engagement. *Handbook of Socialization: Theory and Research*.

[43] Osofsky J. D. (2003). Prevalence of children's exposure to domestic violence and child maltreatment: implications for prevention and intervention. *Clinical Child and Family Psychology Review*.

